# PATTERNS OF USE, RISK FACTORS, AND ATTITUDES OF OLDER ADULT CANNABIS USERS

**DOI:** 10.1093/geroni/igad104.2537

**Published:** 2023-12-21

**Authors:** Elizabeth Anquillare, Adrianna Gallegos, Rachel Thayer

**Affiliations:** University of Colorado Colorado Springs, Colorado Springs, Colorado, United States; University of Colorado Colorado Springs, Colorado Springs, Colorado, United States; University of Colorado Colorado Springs, Colorado Springs, Colorado, United States

## Abstract

While cannabis use continues to increase among older adults (OA), research within this population is limited. This study aimed to describe OA patterns of use, risk factors, and subjective effects of cannabis use. OA cannabis users ages 60-79 (N=17, Mage=68.29, SD=5.61) were 94% White and 59% women, with an average education of 15.94 years (SD=2.38). Participants completed online questionnaires and semi-structured interviews regarding medical and mental health, subjective cognitive functioning, and cannabis use patterns and attitudes. Participants reported M=33.41 years of cannabis use (SD=20.15) with an average age of first use of 20.11 years (SD=10.40). Current use was reported as 4.29 days/week (SD=3.14). Current reasons for use were recreational (n=6), medical (n=2), or both (n=8). The most common medical conditions for which participants reported using cannabis were arthritis/joint pain (n=4) and disordered sleep (n=4). The most commonly reported benefits of cannabis use were relaxation (n=8), improved sleep (n=6), improved mood/anxiety (n=6), cognitive enhancement (n=5), and pain management (n=4). Common drawbacks included subjective cognitive decline (n=7), social hindrance (n=6), and health complications (n=5), whereas 5 participants reported no drawbacks. Most participants (76%) reported discussing cannabis use with their physician, although only 38% of those reported that discussion was helpful. Disconnect between OA cannabis users and their physicians could result in cannabis use that is contraindicated by preexisting medical conditions or medications. Emphasis should be placed on individualized psychoeducation regarding the potential risks/benefits of cannabis use as well as continuing education for physicians regarding cannabis use in OA.

